# Cerebrovascular Events in Aortic Stenosis: From Native Valve Disease to TAVR‐Specific Risk and Prevention

**DOI:** 10.1002/ccd.70660

**Published:** 2026-05-13

**Authors:** Priyanka Boettger, Jamschid Sedighi, Martin Juenemann, Omar Alhaj‐Omar, Kerstin Piayda, Mani Arsalan, Samuel Sossalla, Won‐Keun Kim

**Affiliations:** ^1^ Department of Cardiology, Angiology and Critical Care Medicine Justus‐Liebig University Giessen Germany; ^2^ Department of Neurology and Critical Care Medicine Justus‐Liebig University Giessen Germany

## Abstract

Aortic stenosis (AS) is associated with a heightened burden of cardiovascular comorbidities, atrial fibrillation (AF), and progressive valvular calcification, all of which may contribute to cerebrovascular events across the disease continuum. However, true epidemiologic evidence establishing AS as an independent stroke risk factor remains limited, and much of the contemporary concern regarding stroke has shifted toward interventional management. Transcatheter aortic valve replacement (TAVR) has become the dominant therapy for severe AS, but periprocedural and early postprocedural stroke remain among its most clinically significant complications. This review integrates current knowledge on stroke pathways in native AS, AF, calcific embolization, hemodynamic alterations, and places them in context with procedural mechanisms unique to TAVR. We summarize evidence comparing stroke rates in TAVR versus surgical aortic valve replacement and examine anatomic, procedural, and patient‐level drivers of embolic risk. Building on these mechanisms, we highlight contemporary stroke mitigation strategies including multimodality imaging for preprocedural planning, optimization of access and device selection, cerebral embolic protection devices, antithrombotic therapy tailored to individual indications, and structured postprocedural neurologic and rhythm monitoring. By integrating the natural history of AS with TAVR‐specific embolic pathways, this review provides a comprehensive framework for understanding and minimizing stroke risk in patients across the spectrum of AS.

## 1 Introduction

Aortic stenosis (AS) is the most prevalent valvular disease in older adults, and its progression is typically slow but clinically consequential. While AS is frequently accompanied by cardiovascular comorbidities that may contribute to cerebrovascular events, robust epidemiologic evidence for a direct or independent association with ischemic stroke remains limited. Nevertheless, stroke is an important clinical concern across the disease spectrum, particularly in the context of interventional treatment. With the expanding use of transcatheter aortic valve replacement (TAVR), periprocedural and early postprocedural stroke have emerged as critical outcome determinants. Understanding how native AS pathophysiology, AF, valvular calcification, and procedural factors interact to shape stroke risk is essential for improving patient selection, optimizing procedural planning, and informing prevention strategies.

## 2 Epidemiological Evidence

Evidence suggesting an association between AS and ischemic stroke does exist, although the epidemiologic foundation remains limited. A large Danish retrospective cohort study observed higher stroke incidence in individuals with AS compared with matched controls (30.4 vs. 13.3 per 1000 person‐years), corresponding to a hazard ratio of 1.31 (95% CI, 1.28–1.34) [1]. While this represents the strongest population‐level signal to date, residual confounding from age, AF, and vascular comorbidities cannot be excluded. Earlier population‐based analyses by Boone and by Petty similarly reported increased cerebrovascular events among patients with clinically diagnosed AS, but these studies relied on older diagnostic definitions and lacked comprehensive adjustment for vascular risk factors [2, 3]. Taken together, these findings indicate that AS may contribute to an elevated cerebrovascular risk profile, although current evidence is insufficient to establish a clear independent causal relationship.

## 3 Pathophysiological Mechanisms of Stroke in Aortic Stenosis

**Figure 1:**
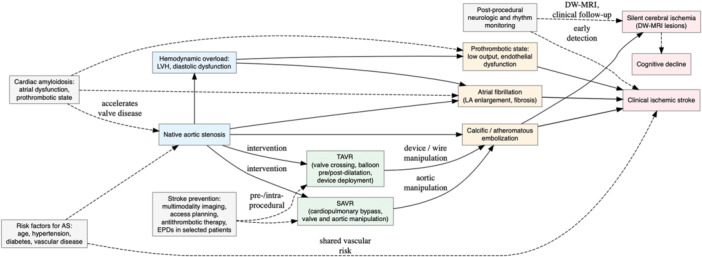
Mechanisms linking AS, TAVR, and cerebrovascular events. This diagram summarizes key pathways contributing to cerebrovascular events across the AS spectrum. Cardiovascular risk factors promote the development of native AS and independently increase stroke susceptibility. Native AS leads to hemodynamic overload, atrial enlargement, AF, and a prothrombotic state, while valvular calcification and cardiac amyloidosis further amplify embolic and thrombotic risk. Interventional treatment adds procedure‐related mechanisms: SAVR involves cardiopulmonary bypass and aortic manipulation, whereas TAVR introduces wire and device manipulation. Both can cause calcific or atheromatous embolization. These mechanisms contribute to clinical ischemic stroke and silent cerebral ischemia, the latter potentially progressing to cognitive decline. Preventive strategies, including multimodality imaging, optimized access, tailored antithrombotic therapy, selective embolic protection, and structured post‐procedural monitoring, act along the entire care pathway to reduce stroke risk. This figure is an original illustration created by the authors, synthesizing established pathophysiological and procedural mechanisms as described in prior studies [4, 5, 6, 7, 8, 9, 10, 11, 12, 13, 14, 15, 16, 17, 18, 19, 20, 21, 22, 23, 24, 25, 26, 27, 28, 29, 30, 31, 32, 33, 34, 35, 36, 37, 38, 39, 40, 41, 42, 43, 44, 45, 46, 47, 48, 49, 50, 51, 52, 53, 54, 55, 56, 57, 58, 59, 60, 61, 62, 63, 64, 65, 66]. [Color figure can be viewed at wileyonlinelibrary.com]

Existing observational data suggest a possible increase in cerebrovascular risk among individuals with AS, but the current evidence base is limited and does not establish AS as an independent causal risk factor. Much of the observed association is likely influenced by shared cardiovascular comorbidities such as hypertension, hyperlipidemia, diabetes, and advanced age, which contribute to systemic atherosclerosis and vascular susceptibility (Figure 1).

### 3.1 Atrial Fibrillation

AF is common in advanced AS due to left atrial enlargement and fibrosis. The chronic pressure overload from AS leads to left ventricular hypertrophy and diastolic dysfunction, which in turn causes left atrial enlargement. This structural remodeling and fibrosis create a substrate for AF, significantly increasing the risk of thromboembolic events, including stroke. The American College of Cardiology highlights that AF is a major risk factor for stroke in patients with AS, necessitating careful monitoring and management [5].

### 3.2 Calcific and Atheromatous Embolization

Calcific and atheromatous debris from the stenotic aortic valve can embolize to cerebral arteries, leading to ischemic stroke. Manipulation of the calcified valve during procedures such as TAVR can dislodge these particles, increasing the risk of embolic events. Likewise, embolic strokes are a significant concern during and after valve replacement procedures due to the potential for debris mobilization [7, 8, 9, 10].

### 3.3 Hemodynamic Alterations and Prothrombotic State

Chronic pressure overload in AS results in left ventricular hypertrophy and diastolic dysfunction. These hemodynamic changes predispose patients to atrial enlargement and thrombogenesis. The increased left ventricular mass and afterload lead to left ventricular systolic dysfunction and eventually to a reduced ejection fraction (HFrEF).

HFrEF and altered diastolic filling pressures both contribute to a prothrombotic state, further elevating the risk of stroke. The American Heart Association emphasizes the role of these hemodynamic alterations in the pathogenesis of stroke in AS patients. In summary, the pathophysiological mechanisms of stroke in AS include AF due to left atrial enlargement and fibrosis, embolization of calcific and atheromatous debris, and hemodynamic changes leading to left ventricular hypertrophy and diastolic dysfunction. These factors collectively contribute to the increased stroke risk in patients with AS, underscoring the importance of comprehensive management strategies to reduce this risk.

### 3.4 Special Conditions: Cardiac Amyloidosis

Cardiac amyloidosis, particularly transthyretin amyloidosis (ATTR) and light‐chain amyloidosis (AL), can lead to AS due to amyloid deposits in the aortic valve, causing thickening and calcification [11]. This combination of conditions is more prevalent in elderly patients. AS itself increases stroke risk while amyloidosis further exacerbates this through atrial dysfunction, a prothrombotic state, and endothelial damage. This combination creates a high‐risk scenario for cerebrovascular events [12].

## 4 Stroke Risk After Aortic Valve Intervention

### 4.1 Stroke Risk in Surgical Aortic Valve Replacement (SAVR) Versus Transcatheter Aortic Valve Replacement (TAVR)

**Figure 2:**
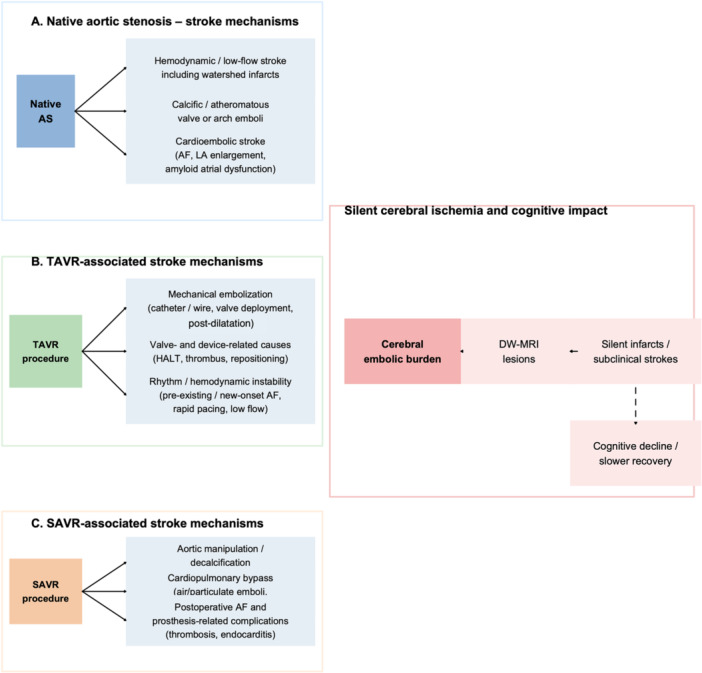
Stroke mechanisms. Mechanistic pathways of stroke in native AS (Panel A), transcatheter aortic valve replacement (Panel B), and surgical aortic valve replacement (Panel C). The right panel outlines the trajectory of silent cerebral ischemia, from procedural embolic burden to DW‐MRI lesions and silent infarcts, with potential implications for cognitive recovery. This figure is an original illustration created by the authors, synthesizing established mechanisms of cerebrovascular injury in aortic stenosis and valve interventions as described in prior studies [5, 6, 7, 26]. [Color figure can be viewed at wileyonlinelibrary.com]

The risk of stroke after surgical aortic valve replacement (SAVR) is about 1.5% in patients with AS (AS), increasing to 2%–4% in high‐risk individuals [13, 14]. This is largely due to the manipulation of the calcified valve and the use of cardiopulmonary bypass, which can trigger embolic events [8, 15]. TAVR is associated with a stroke risk ranging from 0.6% to 6%, depending on patient risk profiles, procedural factors, and timing of outcome assessment [16, 17]. For instance, the PARTNER 3 trial reported a 30‐day stroke rate of 0.6% among low‐risk patients treated with the Sapien 3 valve [18]. In contrast, higher‐risk populations or early‐generation valves have shown stroke rates closer to 4%–6% within 30 days. In high‐risk patients or those undergoing combined procedures, stroke risk after SAVR may rise to 4% [18, 19, 20]. The SURTAVR trial showed a lower early stroke rate with TAVR (3.3% vs. 5.4%; *p* = 0.031) [19], supported by a meta‐analysis confirming reduced stroke risk (HR: 0.81; 95% CI: 0.68–0.98; *p* = 0.028) [20]. Both procedures frequently lead to subclinical cerebral lesions. DW‐MRI scans show new ischemic lesions in 68%–98% of TAVR patients—often without immediate symptoms but potentially linked to cognitive decline and slower recovery [12, 13]. A study by Alassar et al. found ischemic lesions in 76% of TAVR and 71% of SAVR patients 6 days post‐procedure, with no significant difference (*p* = 0.69) [14]. AF substantially influences the interpretation of stroke risk in both SAVR and TAVR [21, 22]. AF is common in advanced AS due to chronic pressure overload, left atrial enlargement, and fibrosis, and new‐onset AF frequently occurs after valve intervention [23]. This dual role as a pre‐existing arrhythmia and a procedure‐related complication acts as a major mediator and confounder of periprocedural stroke risk [24]. As highlighted in recent analyses, AF burden, timing, and management significantly modify cerebrovascular outcomes across the AS–TAVR continuum, and must be considered when comparing stroke rates between SAVR and TAVR [25] (Figure 2).

Early intervention may be beneficial, as demonstrated in the EARLY TAVR study, where patients with asymptomatic severe AS who underwent early TAVR had lower rates of the composite endpoint of death, stroke, and cardiovascular hospitalization compared to those managed with clinical surveillance (HR: 0.50; 95% CI: 0.40–0.63; *p* < 0.001). However, no statistically significant reduction was observed in mortality or stroke when assessed individually. The favorable results of the trial were primarily driven by reductions in cardiovascular hospitalization [15]. While the ACC/AHA guidelines highlight TAVR's lower risk of stroke, major bleeding, and AF in elderly and high‐risk patients [3], some studies challenge this view. The NOTION trial found similar stroke rates at 8 years (8.3% vs. 9.1%; *p* = 0.90) [17], and Shah et al. reported no significant difference at 30 days (2.7% vs. 3.1%; *p* = 0.08) or 1 year (5.0% vs. 4.6%; *p* = 0.96) [16]. TAVR offers clear advantages, particularly for elderly or high‐risk patients, by avoiding open‐heart surgery, reducing aortic manipulation, and shortening the procedure time. This helps lower the risk of embolic events and neurological complications. However, the choice between TAVR and SAVR should be based on individual patient factors, including surgical risk, anatomy, and personal preference. As TAVR expands to lower‐risk patients, ongoing research will be essential in assessing its long‐term impact on stroke risk and overall outcomes. While TAVR appears to offer a marginal advantage in stroke prevention, the optimal treatment strategy should be guided by an individualized, patient‐centered approach.

### 4.2 TAVR in Bicuspid Versus Tricuspid Aortic Valve Anatomy

The data on this topic is conflicting. On one hand there are studies that have demonstrated an increased stroke risk for patients undergoing TAVR with a BAV compared to TAV [27]. For instance, a meta‐analysis by Saeed Al‐Asad et al. found that the rate of 30‐day stroke was higher in patients with BAV who underwent TAVR compared to those with TAV (odds ratio [OR] 1.24, 95% confidence interval [CI] 1.08–1.43, *p* < 0.05) [28].

**Figure 3:**
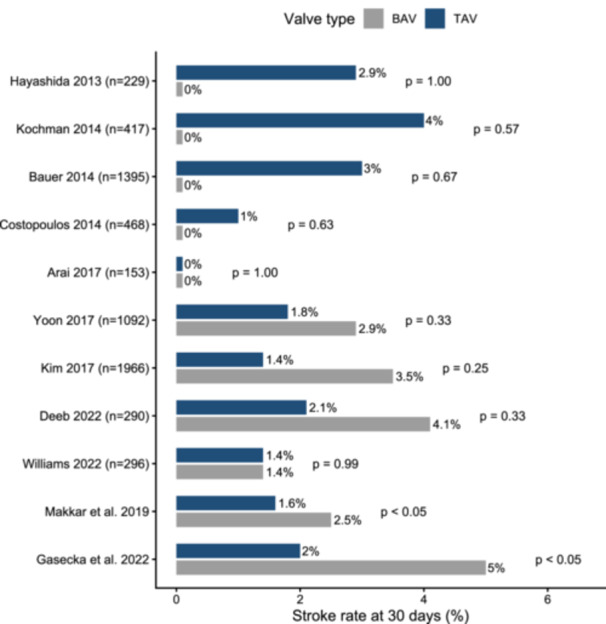
Thirty‐day stroke risk after TAVR in bicuspid versus tricuspid aortic valve morphology. This figure displays 30‐day clinical stroke rates in patients undergoing transcatheter aortic valve replacement (TAVR) with bicuspid (BAV) versus tricuspid (TAV) aortic valve morphology across major observational studies. Absolute event rates are low, particularly in earlier cohorts, and many single‐center or registry studies show no statistically significant differences between BAV and TAV. However, selected large contemporary studies, including Makkar et al. and Gasecka et al., report higher early stroke rates or neurological complications in BAV, illustrating the conflicting nature of the evidence. Overall, the figure highlights substantial heterogeneity across studies, reflecting differences in patient selection, anatomical complexity, device generation, and operator experience. [Color figure can be viewed at wileyonlinelibrary.com]

Similarly, Makkar et al. reported a higher 30‐day stroke rate in patients with BAV (2.5%) compared to those with TAV (1.6%) (hazard ratio [HR] 1.57, 95% CI 1.06–2.33) [29, 30]. A high rate of neurological complications (5%) in patients with BAV‐TAVR has also been demonstrated by Gasecka et al [31]. On the other hand there are multiple studies showing a lower or no stroke risk in BAV‐TAVR [32, 33, 34, 35]. It is important to note that mild neurological deficits may have gone unnoticed by cardiologists. Additionally some of these studies show a significantly higher rate of mortality or cardiovascular events in general for BAV‐TAVR. In the study by Costopoulos et al. (2014) the 30‐day mortality rate was higher in the BAV group (14.2% vs. 3.6%) [33]. Bicuspid aortic valve presents anatomical complexities that may increase procedural challenges and embolic risk. However, evidence remains balanced, with studies reporting elevated, reduced, and unchanged stroke risk, underscoring the need for individualized risk assessment (Figure 3).

### 4.3 Stroke Risk in TAVR for Mixed Aortic Valve Disease

The stroke risk associated with TAVR in aortic regurgitation is relatively low but not negligible. In patients with combined aortic valve disease, including both AS and aortic regurgitation, the stroke risk appears comparable to that in isolated AS. Grant et al. reported a postoperative ischemic stroke incidence of 2% in mixed aortic valve disease, significantly lower than the 5.7% observed in pure AS [36], suggesting that concomitant aortic regurgitation does not necessarily increase stroke risk.

Similarly, Yousef et al. found no significant difference in stroke rates between patients with severe AS alone and those with severe AS combined with moderate or severe aortic regurgitation [37]. However, Nuis et al. identified baseline aortic regurgitation grade III or higher as an independent predictor of stroke, with an odds ratio of 3.2 (95% confidence interval: 1.1–9.3), indicating a modestly elevated risk in this subgroup [38]. In the subgroup of patients who develop symptomatic and severe aortic regurgitation after undergoing SAVR in the past, a similar 30‐day stroke rate of 2% has been reported [37].

Overall, stroke rates in mixed aortic valve disease undergoing TAVR do not appear significantly higher than in isolated AS and may, in some cases, even be lower.

### 4.4 Stroke Risk in TAVR for Isolated Aortic Regurgitation

TAVR is well established for AS, but its application in isolated aortic regurgitation remains less defined and presents unique challenges. Although the stroke risk associated with TAVR in this population is relatively low, it is not negligible. Mentias et al. reported a numerically higher stroke risk in patients undergoing TAVR for pure aortic regurgitation compared to surgical aortic valve replacement, with a hazard ratio of 1.65, though this did not reach statistical significance (95% confidence interval, 0.95–2.87; *p* = 0.07 [39]. Huded et al. highlighted procedural challenges, including the absence of a calcified anchoring zone and the dynamic nature of the aortic annulus, which may increase stroke risk [40]. Despite advancements in newer‐generation devices, Yoon et al. noted that stroke remains a concern [41]. Although the overall stroke risk in TAVR for isolated aortic regurgitation is low, procedural complexities and anatomical factors may contribute to a slightly elevated risk.

## 5 Strategies to Minimize Stroke Risk During and After TAVR

Minimizing stroke risk during TAVR involves a combination of procedural strategies, pre‐procedural planning, and the use of adjunctive devices.

### 5.1 Cerebral Embolic Protection Devices: Benefits and Limitations

The use of embolic protection devices (EPDs) during TAVR has been extensively studied to assess their impact on reducing stroke risk in elderly patients with AS (AS). While EPDs have been demonstrated to reducing disabling stroke and ischemic lesion volume, their overall effect on stroke prevention and mortality remains controversial.

Several studies suggest that EPDs reduce the severity of neurological events. A patient‐level pooled analysis by Seeger et al. found that dual‐filter EPD use significantly lowered all‐stroke rates from 5.44% to 1.88% (odds ratio 0.35, 95% CI 0.17–0.72, *p* = 0.0028) and reduced the combined risk of all‐cause mortality or stroke within 72 h post‐TAVR (2.06% vs. 6.00%; OR 0.34, 95% CI 0.17–0.68, *p* = 0.0013) [42]. Similarly, Butala et al., using STS/ACC TVT Registry data, reported a 13% reduction in disabling strokes (relative risk 0.87, 95% CI 0.73–1.00) and a reduction in the odds of in‐hospital disabling stroke (odds ratio 0.79, 95% CI 0.70–0.90) [43]. A meta‐analysis by Testa et al. further supported these findings, showing that EPDs reduced 30‐day stroke risk (odds ratio 0.55, 95% CI 0.31–0.98, *p* = 0.04) and significantly decreased ischemic lesion volume per patient (standardized mean difference −0.52, 95% CI −0.85 to −0.20, *p* = 0.002) [44]. These findings suggest that while EPDs do not eliminate embolic risk, they may limit the extent of cerebral damage, potentially improving clinical outcomes [4, 6].

Despite these benefits, the overall effectiveness of EPDs in stroke prevention remains uncertain. The PROTECTED TAVR trial found no significant reduction in overall periprocedural stroke rates between patients who received EPDs and those who did not (2.3% vs. 2.9%; *p* = 0.30) [6]. Additionally, meta‐analyses indicate that EPD use has not been associated with lower 30‐day mortality [43, 45].

Importantly, the trials that did not demonstrate a reduction in overall periprocedural stroke‐ including PROTECTED TAVR and other randomized evaluations, are all rigorously conducted randomized controlled studies, whereas reports suggesting benefit derive largely from observational registries, in which treatment selection and unmeasured confounding remain unavoidable.

Another limitation is that while EPDs reduce ischemic lesion volume, they do not significantly decrease the number of new cerebral lesions detected on diffusion‐weighted MRI (DW‐MRI), suggesting that some embolization occurs despite their use [26]. The technical challenges and costs associated with EPD deployment also warrant consideration. While the success rate of EPD placement is high (94.5%), device malpositioning or procedural failure can still occur, adding complexity to TAVR procedures. The additional cost of EPDs may not be justified if overall stroke rates and mortality remain unchanged [26, 43].

In summary, in some studies EPDs offer clear benefits in reducing disabling strokes and ischemic lesion volume, yet their lack of impact on overall stroke rates and mortality raises questions about their routine use. Given the technical challenges and cost implications, the decision to use EPDs should be individualized, considering patient‐specific risk factors and anatomical considerations. Further large‐scale randomized trials and meta‐analyses are needed to determine whether the benefits of EPDs justify their widespread adoption in routine TAVR practice (Table 1).

**Table 1:** Summary of different cerebral protection devices and their respective clinical trials.

Device	Coverage/Access site	Sheath/Pore size (mm)/Mechanism	Clinical trials	SD	Number of patients	Clinical findings
Sentinel	2 vessels/radial	6 Fr/140/capture	MISTRAL‐C [44]	RCT	Device: 32, Control: 33	Two disabling strokes in the control arm within 30 days
			CLEAN‐TAVI [40]	RCT	Device: 50, Control: 50	10% of both groups had non‐disabling stroke within 7 days
			SENTINEL [45]	RCT	Device safety arm: 123, Imaging: 121, Control: 119	Stroke at 30 days: device = 5.6% versus control = 9.1% (*p *= 0.25) MACCE at 30 days: device = 7.3% versus control = 9.9% (*p *= 0.4)
TriGUARD	3 vessels/femoral	8 Fr/145/deflector	DEFLECT III[46]	RCT	Device: 46, Control: 39	MACCE at 30 days: device = 21.7% versus control = 30.8% (*p *= 0.34) Stroke: device = 2.2% versus control = 5.1% (*p *= 0.46)
			REFLECT I[47]	RCT	Device: 162, Control: 121	Combined primary safety at 30 days: device = 15.9% versus control = 7% (*p *= 0.11)
EMBOL‐X	3 vessels and body/transaortic	17 Fr/120/capture	TAo‐EmbolX [48]	RCT	Device: 14, Control: 16	No neurological events seen
Embrella	2 vessels/radial	6 Fr/100/deflector	PROTAVI‐C Pilot [49]	Non‐RCT	Device: 41, Control: 11	1 TIA and 2 strokes in the device group
Emblok	3 vessels/femoral	11 Fr/100/capture	Latib et al [50]	Single arm	Device: 20	No MACCE at 30 days
Emboliner	3 vessels and body/femoral	10 Fr/150/capture	SafePass I, II [51]	Single arm	Device: 20	No MACCE at 30 days
ProtEmbo	3 vessels/left radial	6 Fr/60/deflector	PROTEMBO SF [52]	Single arm	Device: 41	MACCE at 30 days: 8% with device

Abbreviations: MACCE, major adverse cardiovascular and cerebrovascular events; RCT, randomized controlled trials; SD, study design; TAVR, transcatheter aortic valve implantation; TIA, transient ischemic attack.

### 5.2 Access Route

The relationship between the access route for TAVR and the risk of stroke is significant.

Studies have shown that the choice of vascular access can influence the incidence of stroke post‐TAVR. Specifically, supra‐aortic access (such as transcarotid, transapical or axillar) is associated with a higher risk of stroke compared to transfemoral access. Ricco et al. found that supra‐aortic access had an odds ratio (OR) of 9.00 (95% CI: 2.95–27.44; *p* = 0.001) for early stroke compared to femoral access [55]. A meta‐analysis by Usman et al. evaluated transcarotid TAVR and reported a stroke/transient ischemic attack (TIA) rate of 3.4% at 30 days, which is comparable to other access routes but still highlights the risk associated with non‐femoral approaches [56]. Additionally, Athappan et al. found no significant difference in 30‐day stroke rates between transfemoral and transapical approaches in their meta‐analysis but noted that the overall stroke risk remains a concern with both approaches [57].

In summary, transfemoral access is generally associated with a lower risk of stroke compared to supra‐aortic access routes during TAVR.

### 5.3 Pre‐Procedural Imaging and Planning

Pre‐procedural imaging is essential in TAVR to minimize stroke risk in elderly AS patients. Multimodal imaging, particularly MDCT, is the gold standard for assessing annulus size, calcification, and vascular access. Accurate measurements help optimize valve selection, reducing the risk of paravalvular regurgitation and embolic events [58, 59]. The American College of Radiology highlights CTA with IV contrast as essential for assessing the supravalvular aorta and vascular access, aiding in complication prevention and procedural planning [60]. It helps identify challenges like tortuous or calcified vessels, reducing embolic stroke risk [61]. Pre‐procedural imaging identifies high‐risk features like low coronary ostia, small sinuses of Valsalva, and aortic arch calcification, which increase stroke risk in TAVR [62].

This allows the heart team to identify anatomic features that may heighten embolic risk and to integrate appropriate mitigation strategies into procedural planning [63]. In summary, thorough imaging assessment and careful pre‐procedural planning remain central to minimizing stroke risk and achieving optimal outcomes in TAVR candidates.

### 5.4 Intra‐Procedural Strategies

Minimizing manipulation of calcified valves and the aortic arch during TAVR is essential to reduce stroke risk. Operators should use careful catheter techniques, limit wire exchanges, and ensure precise valve positioning to prevent embolization. In addition to procedural technique, anatomic factors may also contribute to embolic risk. Huded et al. proposed that the combination of a calcified anchoring zone and a dynamic annular morphology can predispose patients to cerebral embolism, particularly in anatomically complex cases [40].

The American Society of Echocardiography emphasizes controlled deployment, while rapid ventricular pacing during balloon‐expandable valve placement, such as with the Edwards SAPIEN 3, helps stabilize the valve and further reduce embolic risk [59]. Computational predictive modeling has also been shown to be effective in pre‐procedural planning to avoid complications such as coronary artery occlusion, which can indirectly reduce the risk of stroke by ensuring optimal valve positioning and deployment [64]. However, this technique is not broadly available and hence has not entered widespread clinical practice.

**Figure 4:**
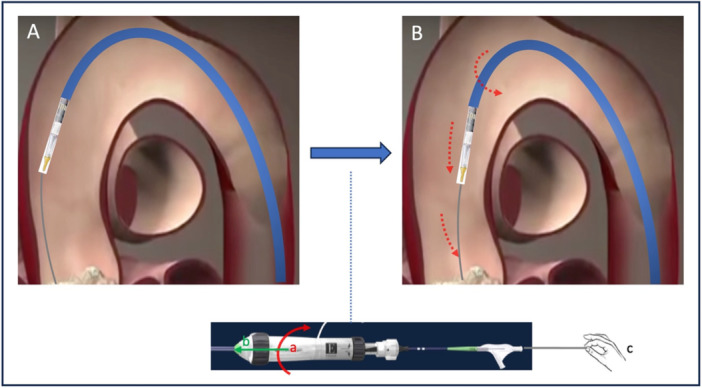
Controlled catheter manipulation in the aortic arch to reduce embolic risk during transfemoral TAVR. (A) Initial position of the delivery system with the catheter running along the outer curvature of the aortic arch. (B) Steering maneuver to bring the catheter into a more central, atraumatic course by gentle rotation and adjustment of the tip. The handle (a) allows controlled rotation/torque of the catheter, (b) indicates deflection and orientation of the distal tip, and (c) denotes fine advancement or withdrawal of the shaft by hand. This combination of movements helps to minimize scraping of calcified aortic wall and potential embolization. This figure is reproduced with permission from PCRonline [66]. [Color figure can be viewed at wileyonlinelibrary.com]

Shorter procedural times and precise catheter control further minimize embolic risk (Figure 4). Device‐based cerebral protection strategies and their uncertain impact on clinical stroke outcomes are discussed in detail in the section on EDPs. Overall, reducing valve manipulation, ensuring controlled deployment are key strategies for stroke prevention in TAVR [65].

### 5.5 Antithrombotic Management

Antithrombotic management is a vital component of stroke prevention during TAVR. The ACC/AHA guidelines suggest that in patients without an indication for long‐term anticoagulation, single antiplatelet therapy (SAPT) with aspirin is generally preferred over dual antiplatelet therapy (DAPT) [5]. This approach is supported by multiple studies, including the POPULAR‐TAVR trial, which demonstrated that SAPT was associated with fewer bleeding complications without a significant increase in thrombotic events compared to DAPT [67] (Central illustration 1).

**Central illustration 1:**
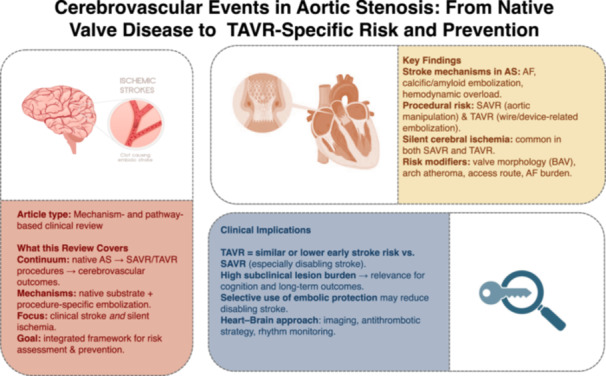
Aortic stenosis increases cerebrovascular vulnerability through atrial fibrillation, calcific embolization, and hemodynamic overload, while SAVR and TAVR add procedure‐specific embolic risks. Integrating native‐disease mechanisms with TAVR‐specific pathways enables targeted imaging, procedural planning, and antithrombotic strategies to reduce clinical stroke and silent cerebral injury across the AS continuum. [Color figure can be viewed at wileyonlinelibrary.com]

For patients requiring long‐term anticoagulation, such as those with AF, oral anticoagulation (OAC) alone is recommended. While VKAs effectively reduce thromboembolic events, DOACs may be considered if appropriate. The GALILEO trial showed increased bleeding risk with a rivaroxaban + ASS strategy, emphasizing the need for careful DOAC selection [68].

In high‐risk cases like recent acute coronary syndrome or complex stenting, short‐term DAPT may be needed to balance bleeding and thrombosis risks. ESC guidelines stress individualized therapy. Overall, SAPT is preferred without long‐term anticoagulation needs, while OAC is recommended for AF, with DAPT reserved for select high‐risk patients [69].

### 5.6 Post‐Procedural Monitoring

Post‐procedural monitoring after TAVR is crucial for the early detection of neurological and rhythm‐related complications that influence short‐term and long‐term outcomes. Immediate post‐procedural observation remains essential, as conduction disturbances, new‐onset AF, and early cerebrovascular events typically occur within the first 2 days.

Rhythm surveillance after TAVR is now guided by a risk‐stratified strategy, as reflected in the 2025 ESC/EACTS [70] and the 2023 AHA/ACC [70] Valvular Heart Disease Guidelines. Both recommend short in‐hospital telemetry, typically 24–48 h, with extended ambulatory ECG monitoring reserved for patients who develop new conduction abnormalities—particularly new left bundle‐branch block, PR/QRS prolongation, or dynamic changes suggestive of delayed high‐grade atrioventricular block. This aligns with contemporary fast‐track TAVR pathways, in which most patients are discharged within 1–2 days, and reflects the shift away from a universal 3‐day monitoring paradigm. New‐onset AF remains common during the first 30 days and is strongly associated with periprocedural stroke, supporting selective prolonged rhythm monitoring in high‐risk subgroups [17].

Neurological monitoring should follow structured early assessment protocols. Frequent neurological examinations during the first 24 h facilitate timely identification of evolving deficits, and early neurointervention, particularly mechanical thrombectomy, has been associated with improved outcomes in TAVR‐related ischemic stroke in multicenter registries [71, 72, 73]. Close coordination between cardiology and neurology teams is therefore integral to optimal post‐procedural care [74, 75].

In summary, modern post‐TAVR monitoring relies on short, structured in‐hospital observation, selective ambulatory rhythm surveillance, and protocolized neurological assessment, consistent with the most recent ESC/AHA guidelines. This risk‐adapted approach supports safe early discharge while maintaining vigilance for conduction disturbances and cerebrovascular events.

## 6 Key Messages

- AS (AS) is a significant yet underrecognized risk factor for ischemic stroke, driven by AF, embolization of calcific debris, and hemodynamic changes leading to left ventricular hypertrophy and atrial enlargement.
- Surgical and transcatheter interventions for AS, including SAVR and TAVR, carry an inherent stroke risk, primarily due to procedural manipulation of calcified valves, underscoring the need for careful patient selection and risk mitigation strategies.
- TAVR has demonstrated a lower early stroke risk compared to SAVR, with emerging evidence supporting the role of embolic protection devices, advanced imaging, and optimized antithrombotic therapy to further reduce embolic complications.
- Risk stratification and individualized management are crucial for stroke prevention in AS patients, particularly in younger individuals and those undergoing intervention, necessitating a multidisciplinary approach integrating cardiology, neurology, and interventional expertise.
- Future research should focus on refining stroke prevention strategies and optimizing procedural techniques, including evaluating the role of early TAVR in moderate AS, to improve long‐term cerebrovascular outcomes in this high‐risk population.

## 7 Conclusion

The relationship between AS and ischemic stroke is complex, driven by valvular dysfunction, hemodynamic changes, and thromboembolic mechanisms. AF, calcific embolization, and left ventricular hypertrophy contribute to elevated stroke risk, particularly in younger patients and those undergoing intervention. TAVR has transformed AS management, offering a lower stroke risk than SAVR due to its minimally invasive approach and avoidance of cardiopulmonary bypass. Advances such as EDPs, imaging‐guided planning, and optimized antithrombotic strategies further enhance safety. Despite progress, stroke remains a key concern, necessitating comprehensive risk stratification and multidisciplinary collaboration. This review underscores the need for continued research to refine stroke prevention strategies and improve long‐term outcomes in AS patients.

## Conflicts of Interest

The authors declare no conflicts of interest.

## Data Availability

The data that support the findings of this study are available from the corresponding author upon reasonable request.
